# Breaking free from prognostic paralysis in chronic advanced diseases

**DOI:** 10.1017/S1478951525100254

**Published:** 2025-06-23

**Authors:** Isabel Galriça Neto, Teresa Sarmento, Eduardo Bruera

**Affiliations:** 1 Head of Palliative Care Department, Hospital da Luz-Lisboa, Lisbon, Portugal; 2 Catolica Católica Medical School, Faculdade de Medicina de Lisboa, Lisbon, Portugal; 3 ULSNE, Bragança, Portugal; 4 ULSTMAD, Vila Real, Portugal; 5 APCP, Lisbon, Portugal; 6 Department of Palliative Care, Rehabilitation, and Integrative Medicine, University of Texas MD Anderson Cancer Center, Houston, TX, USA

## Introduction

In recent decades, we have witnessed important developments in new and revolutionary systemic cancer treatments, particularly in terms of immunotherapy and targeted therapies, which have made treating incurable solid cancers more successful but also more challenging and unpredictable. Updated treatment illness trajectories for patients with incurable solid cancers include major temporary improvement, long-term ongoing response, and rapid decline with death (Geijteman et al. 2024).

Similar trends are observed in the management of other chronic, incurable diseases such as COPD, heart failure, or degenerative neurological diseases (Chang and Wong, 2018; Epiphaniou et al. 2014a). Patients may experience longer disease trajectories with significant temporary improvements, leading to increased prognostic uncertainty for patients, caregivers, and clinicians.

Chronic illnesses often present unpredictable trajectories, making it challenging for healthcare professionals to determine when to initiate palliative care (PC). While there is increasing consensus that patients’ needs – namely, the symptomatic burden and the existential aspects of suffering – should take precedence over prognosis in PC referrals, the complex decision-making process in clinical practice remains heavily focused on prognostic indicators rather than patient-centered care (Epiphaniou et al, 2014b; Kobda-Ceh et al. 2025; Rajnoveanu et al. 2020).

In clinical practice, it may be difficult for clinicians to recognize the signs of terminality and even proximity to death, leading to an increase in acute care during the last year of life and persistent late integration of PC (Martins-Branco et al. 2020; Hawkins et al. 2024; Hu et al. 2025).

Reflecting on this increasingly frequent reality in the field of end-of-life care, we have come across the concept of prognostic paralysis, and we want to draw your attention to it.

## What is prognostic paralysis?

The term “prognostic paralysis” was first documented in a 2005 article (Murray et al., 2005) in the *British Medical Journal* (BMJ) titled “Palliative care in chronic illness: we need to move from prognostic paralysis to active total care.” Since then, “prognostic paralysis” has been referenced in various medical discussions, particularly concerning chronic illnesses with unpredictable progressions, such as chronic obstructive pulmonary disease (COPD). For example, a 2013 study (Benzo, 2013) highlighted the challenges clinicians face in predicting end-of-life in COPD, noting that this uncertainty often leads to “prognostic paralysis,” thereby delaying essential PC discussions.

The reluctance to engage in goals-of-care discussions and PC referrals often arises from clinicians’ concerns about causing distress or diminishing hope (Corcoran and Kluger 2023; Hui et al, 2015). Such hesitation can result in clear inadequate care planning and clinical support for patients nearing the end of life (Corcoran and Kluger, 2023; Hasegawa et al. 2024).

Over the past decades, since Dame Cicely Saunders presented “hospice care” in the 1960s, and Balfour Mount later coined the term “palliative care” in the 1970s, the field has evolved from treating patients only at the end of life into a highly specialized discipline focused on delivering appropriate care to patients with life-limiting illnesses throughout the disease trajectory. PC today is much more than a “death sentence,” and findings from multiple studies indicate that integrating PC early in the disease trajectory can result in improvements in quality of life, symptom control, patient and caregiver satisfaction, decision-making capacity, quality of end-of-life care, as well as survival and costs of care (Hui and Bruera, 2016a; Kodba-Čeh H, 2025).

Our concern as clinicians in PC and oncology, observing every year many patients who receive new systemic treatments in a situation of advanced and incurable disease, is precisely to re-observe a phenomenon that seems cyclical to us, perhaps too ingrained in human nature – the denial of our terminality and the inevitability of death (Breitbart, 2017). With each new progress in medicine, we stop talking about it, about deathand the reluctance to acknowledge our mortality increases. In this still illusion of a “cure for all,” those who suffer most are the sick and caregivers, who are deprived of the care most appropriate to their needs and furthest from their values. This approach can cause unnecessary suffering and place a significant financial burden on healthcare systems, especially during the last year of life (Hawkins et al. 2024; Murray and Amblàs, 2021).

The over-medicalization of death (Clark, 2002; Hawkins et al. 2024; Murray and Amblàs, 2021) is a critical issue related to this concept of paralysis. Ivan Dominic Illich, an Austrian philosopher, in the mid-’70s, and then Clark in 2002, drew attention to a certain “sense of being in a state of ‘total war’ against death at all stages of the life cycle.” Breitbart (2017) wrote beautifully about the inevitability of death, death anxiety, and the concept of “middle knowledge.”

Being aware of the challenges faced by all doctors with good modern therapies, we intend with this article to draw attention to the relevance of this “new-old” entity in clinical practice – prognosis paralysis – and to invite others to reflect on it.

## Further steps

Inaction in the face of uncertainty is a clinical decision with its own risks. How to overcome this phenomenon that has a high impact on the lives of patients and paralyzes good end-of-life care? Here are some clues for reflection.

Clinicians often grapple with the difficulty of predicting life expectancy, but avoiding these discussions does a disservice to patients. Attempts to focus on the accuracy of prognostication will only reinforce the perception that PC is for dying patients. The discussion of prognosis goes beyond the discussion of life expectancy and covers issues such as quality of life after treatments, the management of uncertainty and incurability, and the remaining functional prognosis (Baxter et al, 2024). By embracing early and honest conversations, not only about prognosis but also on patient needs, well-being, values, and goals of care, healthcare providers can empower patients to make informed decisions, ensuring their final months are lived with dignity and comfort (Baxter et al, 2024; Hasegawa, 2024).

To overcome prognostic paralysis and enhance care for patients with chronic illnesses, it is recommended that healthcare providers adopt a proactive approach by focusing on the patient’s current needs rather than waiting for precise prognostic indicators (Boyd and Murray, 2010; Bruera and Hui, 2010; Hui et al. 2016b). This strategy involves promoting proactive anticipatory care planning from the time of diagnosis, planning holistic care that integrates palliative care measures alongside ongoing treatments, ensuring that patients receive comprehensive support throughout their illness transitions, and enabling them to make informed decisions (Blay et al. 2017). Such an approach not only improves care coordination but also significantly enhances the quality of life for both patients and their caregivers.

Summarizing, we suggest some strategies to overcome prognostic paralysis (Box 1).

Box 1 – Main strategies to overcome prognostic paralysis

**Category Action Description**

Education and Training: Provide healthcare professionals with training in communication skills and serious illness conversations to facilitate discussions about prognosis and end-of-life care.

Early Integration of PC: Incorporate PC principles early in the disease journey, focusing on needs assessment, symptom management, psychosocial support, and advance care planning. Promote patient-centered care and shared-care between other specialties and PC doctors.

Proactive Anticipatory Care Planning: Encourage discussions about future care preferences, potential scenarios, and goals of care, allowing patients and families to make informed decisions. Promote care coordination between different resources of care (hospital/community).

Patient and Family Engagement Involve patients and their families in care planning to ensure that care aligns with their goals and values. Discuss goals and preferences with patients and caregivers, ideally at the time of diagnosis and throughout the course of the illness, especially given the prognostic uncertainty of systemic anticancer treatment or other therapies.

Research on End-of-Life Care Encourage health professionals’ reflection, promote qualitative and quantitative research on patterns and intensity of care at the end-of-life (last 6 to 12 months of life), comparing patient-reported outcomes and quality of life.

## Conclusion

Breaking free from prognostic paralysis requires a new attitude, a cultural shift in medicine – one that prioritizes holistic care over aggressive interventions at all costs. The time to act is now. We must work together and replace uncertainty-driven inaction with compassionate, anticipatory care that aligns with the true needs of those facing the end of life and allowing them to be the leading actors in their own journey.
